# Remodeling the tumor immune microenvironment with oncolytic viruses expressing miRNAs

**DOI:** 10.3389/fimmu.2022.1071223

**Published:** 2023-01-04

**Authors:** Guillaume St-Cyr, Daphné Penarroya, Lauren Daniel, Hugo Giguère, Almohanad A. Alkayyal, Lee-Hwa Tai

**Affiliations:** ^1^ Department of Immunology and Cell Biology, Université de Sherbrooke, Sherbrooke, QC, Canada; ^2^ Department of Medical Laboratory Technology, Tabuk, Saudi Arabia; ^3^ Immunology Research Program, King Abdullah International Medical Research Center, Riyadh, Saudi Arabia; ^4^ Research Centre of the Centre Hospitalier de l'Universite de Sherbrooke (CHUS), Sherbrooke, QC, Canada

**Keywords:** tumor microenvironment, oncolytic viruses, miRNAs, immunotherapies, immune cells

## Abstract

MiRNAs (miRNA, miR) play important functions in the tumor microenvironment (TME) by silencing gene expression through RNA interference. They are involved in regulating both tumor progression and tumor suppression. The pathways involved in miRNA processing and the miRNAs themselves are dysregulated in cancer. Consequently, they have become attractive therapeutic targets as underscored by the plethora of miRNA-based therapies currently in pre-clinical and clinical studies. It has been shown that miRNAs can be used to improve oncolytic viruses (OVs) and enable superior viral oncolysis, tumor suppression and immune modulation. In these cases, miRNAs are empirically selected to improve viral oncolysis, which translates into decreased tumor growth in multiple murine models. While this infectious process is critical to OV therapy, optimal immunomodulation is crucial for the establishment of a targeted and durable effect, resulting in cancer eradication. Through numerous mechanisms, OVs elicit a strong antitumor immune response that can also be further improved by miRNAs. They are known to regulate components of the immune TME and promote effector functions, antigen presentation, phenotypical polarization, and varying levels of immunosuppression. Reciprocally, OVs have the power to overcome the limitations encountered in canonical miRNA-based therapies. They deliver therapeutic payloads directly into the TME and facilitate their amplification through selective tumoral tropism and abundant viral replication. This way, off-target effects can be minimized. This review will explore the ways in which miRNAs can synergistically enhance OV immunotherapy to provide the basis for future therapeutics based on this versatile combination platform.

## Introduction

MiRNAs are molecular regulators of cellular processes and have been implicated in both the suppression of tumors and underlying processes in tumorigenesis and tumor progression. Tumor suppressor miRNAs are often dysregulated in cancer, while tumor promoting miRNAs, or oncomirs, are overexpressed (1). This leads to the establishment of two different therapeutic approaches: replacement and inhibition.

Therapies that target tumor-suppressor miRNAs or oncomirs have emerged towards clinical trials (2). The success of these miRNA therapies hinges on several factors including their effective delivery into the TME. However, most currently used vectors lack the tumor specificity required for optimal *in situ* activity, which leads to undesirable off-target effects and diminished therapeutic efficiency (3). Moreover, the dysregulation of the miRNA biogenesis pathway and of the miRNAs themselves found in cancer, combined with the tumorigenic corruption of cellular processes, can present formidable obstacles for miRNA therapeutics (4). This highlights the importance of selecting the appropriate miRNAs to replenish or suppress and ensure tumor selectivity. Together, these limitations and considerations suggest the need for a better delivery vector with properties that can enhance the effect of miRNA therapeutics.

One potential delivery vector is OVs, which are a class of immunotherapeutic agents capable of selectively lysing cancer cells, while simultaneously eliciting an antitumor immune response (5). They can also be used to overcome challenges in miRNA-based therapies. OVs have been engineered to express miRNAs and deliver them directly in the TME through tumor tropism (6). This enables precise regulation of tumor suppressor gene and oncogene expression in tumor cells, while limiting off-target harmful effects. OVs can therefore, potentiate the development of miRNA therapeutics.

Both miRNAs and OVs, alone, have proven their ability to modulate the immune compartment of the TME to promote an antitumor immune response. However, their synergistic combination has yet to be thoroughly investigated. This review will explore the current state of viral oncotherapy enhanced with miRNAs to improve immune TME-remodeling.

## MiRNA biogenesis and dysregulation in cancer

MiRNAs are small non-coding RNA molecules involved in post-transcriptional gene expression regulation through RNA silencing. They are initially expressed as primary transcripts (pri-miRNA) and go through multiple processing steps reviewed extensively elsewhere (7). MiRNAs can be divided into two categories. The first includes miRNAs involved in oncogenesis, tumor progression and metastasis, while the second groups those acting as tumor suppressors (1). The pathways involved in miRNA processing and the miRNAs themselves are dysregulated in cancer. The transcription of pri-miRNAs is affected in cancer, as many miRNA genes are in unstable genomic regions, which leads to altered miRNA-expression profiles. In turn, the expression of miRNAs is regulated by tumor-suppressor genes or oncogenes, which are often altered in cancer. Moreover, epigenetic modifications also interfere with the expression of miRNAs in cancer at the genomic level where DNA methylation and histone modification cooperate to supress miRNA expression. Overall, the ability of miRNAs to regulate multiple tumor-suppressors or onco-genes is significantly altered in cancer, through multiple distinct mechanisms, which favors the development and progression of tumors (4).

## MiRNAs in the immune TME

The effects of the miRNA dysregulation found in cancer cells contributing to the malignant phenotype and corrupting cellular processes, are contagious. The altered miRNAome is propagated to different cellular compartments within the TME through exosomes that are secreted by cancer cells loaded with oncomirs and depleted of tumor-suppressor miRNAs. This mechanism of communication has been demonstrated to facilitate cancer initiation and progression, the process of epithelial-mesenchymal transition (EMT), metastasis, angiogenesis, and the development of chemoresistance through epigenetic modifications (8). The immune TME is composed of both innate and adaptive immune cells that serve to eradicate or support tumor cells. Anti-tumorigenic immune cells include M1-like macrophages, dendritic cells, and activated natural killer (NK) and CD8+ T cells. However, regulatory immune cells can be observed in this environment, such as myeloid-derived suppressor cells (MDSCs), M2-like macrophages and T regulatory (Tregs) cells that can all have a profound impact on aiding tumors to avoid immune detection (9–11). The immune phenotype of tumors has been described as variations of ‘cold’ tumors, those without immune infiltrate or highly immunosuppressive, and ‘hot’ tumors, those that are inflamed (12, 13).

*CD8+ cytotoxic T cells (CTLs).* MiRNAs play an important role in the induction of T cell suppression. Liu et al. (2019) reported that endoplasmic reticulum stress could stimulate hepatocellular carcinoma (HCC) cells to secrete miR-23a-3p-enriched exosomes. These extracellular vesicles upregulated the expression of AKT and PD-L1 by targeting the phosphatase and tensin homolog, and tumor suppressor gene PTEN. This promoted T cell exhaustion and apoptosis (14). In acute myeloid leukemia (AML), miR-19a-3p packaged into small extracellular vesicles secreted (SEV) from leukemic cells was shown to be internalized by CD8+ T cells and disrupted their function, leading to immune evasion (15). MiRNAs can also stimulate T cell responses. The expression of the miR-183/96/182 cluster (m96cl) was significantly repressed in mesenchymal cells in several different types of cancers including non-small cell lung carcinoma (NSCLC). Ectopic expression of m96cl was shown to inhibit *in vivo* tumor growth and metastasis through IL2-mediated stimulation of CD8+ CTLs, by targeting Foxf2 and Zeb1. Depletion of IL-2 resulted in the loss of m96cl/IL2-activated CD8+ CTLs and recovery of metastatic properties (16). Moreover, it was demonstrated in mouse models of adoptive cell therapy that the antitumor activity of CD8+ CTLs could be further stimulated through the transduction of miR-200c and subsequent repression of Zeb1 (17).

*Immunosuppressive cells.* Tregs and MDSCs are major drivers of immunosuppression in the TME, which promotes tumor growth and progression (18, 19). Both are regulated by miRNAs (20, 21). Ning et al. demonstrated that colorectal cancer cells secreting high levels of miR-208b stimulated the expansion of Tregs by targeting the programmed cell death factor 4 (PDCD4) and favored resistance to oxaliplatin (22). Interestingly, PDCD4 is a confirmed tumor-suppressor gene in multiple types of cancer. Its downregulation has been shown to promote the proliferation of gliomas (23). PDCD4 is also targeted by miR-21, which stimulates the invasion and metastasis of colorectal cancer cells (24). In immune cells, however, PDCD4 appears to promote chronic inflammation. In fact, its multiple roles have been reviewed by Jiang et al., in 2017 (25). A study, highlighting the contribution of MDSC-miRNA interactions in the induction of metastasis following radiotherapy in esophageal squamous cell carcinoma (ESCC), demonstrated that irradiated tumor cells secreted miR-26b-5p-enriched SEVs. These SEVs induced the expansion and activation of MDSCs by targeting PTEN (26). Similarly, miR-1298-5p promotes glioma progression through targeting the known tumor suppressor MSH2 in MDSCs. However, in glioma cells, miR-1298-5p has direct tumor suppressive effects (27).

*DCs.* DCs have a central role in antitumor immunity as they cross-present tumor-associated antigens (TAAs) to T cells, driving a stronger CD8+ T cell response as well as supporting Th1 polarization of CD4+ T cells. However, DCs in the TME are associated with an increased expression of IDO1, an immunosuppressive enzyme that inhibits the proliferation and effector function of T cells and NK cells as well as promoting the differentiation of Tregs. Therefore, depending on the incoming signals, DCs can either favor or hinder tumor progression (28). Many miRNAs have been associated with the development and differentiation of DCs and are implicated in the regulation of their inflammatory response (29). Naturally, miRNAs can also modulate DCs in the TME. For example, Lewis lung carcinoma (LLC) cells secrete miR-21/miR-29-enriched exosomes that, once engulfed by DCs, increase TNF-α and IL-6 production by targeting TLR7/8. This leads to a pro-tumoral inflammatory state involved in tumor growth and metastasis (8). In pancreatic cancer cells, miR-203 is associated with a reduced expression of TLR4 and of key cytokines such as IL-12 and TNF-α. This directly impairs antigen presentation in DCs and enhances tumor growth (30).

*Tumor-associated macrophages (TAMs).* TAMs are categorized in two groups with polarization states favouring an M1- or M2-like phenotype (31). In breast cancer models, activated endothelial cells were shown to deliver several miRNAs, including miR-183-5p, miR-222-3p and miR-142-5p to TAMs through extracellular vesicles. The treatment of breast tumor-bearing mice with exosomes enriched with these three miRNAs induced the polarization of macrophages towards a M2-like phenotype and favored tumor growth (32). This M2 polarization phenomenon has also been associated with the secretion of exosomal miRNAs: miR-21 in head and neck cancer, miR-29a-3p in oral SCC, miR-145 in colorectal cancer, miR-301a-3p in pancreatic ductal adenocarcinoma (PDAC), miR-503 in breast cancer, and miR-1246 in glioma and ovarian cancers (33). In epithelial ovarian cancer, TAMs have demonstrated their ability to skew the Tregs/Th17 ratio by delivering miRNA-enriched exosomes to CD4+ T cells. The extracellular vesicles containing STAT3-targeting miRNAs, including miR-29a-3p and miR-21-5p, downregulated cytokines such as IL-4, TNF-α and IL-6, but upregulated the anti-inflammatory cytokine IL-10, which contributed to the establishment of a pro-tumoral imbalance in the TME (34). Interestingly, high-density lipoprotein (HDL) and low-density lipoprotein (LDL) have also been shown to transport miRNAs from cancer cells to macrophages (35).

*B cells.* Relatively little is known about the function of miRNAs in B cells populating the TME. Recent studies have started to reveal their importance. In ovarian cancer, the transfer of miR-330-3p from B cells to tumor cells in exosomes has been correlated with poor prognosis. Although the primary function of miRNAs is to regulate gene expression post-transcriptionally, the presence of miR-330-3p was detected in the nucleus and shown to interact directly with the promoter region of the junctional adhesion molecule 2 (Jam2) gene and increase its expression. In this manner, B cells were able to induce a mesenchymal phenotypical switch in ovarian tumor cells, leading to enhanced migration and invasion (36). A separate study demonstrated that ectopic expression of the miR-212/132 cluster in B cells can interfere with the development of leukemia, in mice (37). In lymphomas, several miRNAs, expressed in B cells are associated with both pro- and anti-tumorigenic roles (38).

*NK cells.* NK cells have important roles in antitumor immunity and cancer immunosurveillance, as NK cells are specifically equipped with a constellation of activating and inhibitory receptors to detect malignant cells (39). Previously, we have demonstrated and reviewed the importance of NK cells in tumor models of virus-based cancer therapy (40, 41). TGF-β has been shown to be a potent inhibitor of antitumor immunity and exerts its functions through multiple mechanisms including the impairment of NKG2D-mediated cancer immunosurveillance (42). This was shown to be, at least in part, due to the translational repression of NKG2D by miR-1245, a miRNA positively regulated by TGF-β (43). CX3CR1-mediated infiltration of NK cells is diminished by the expression of miR-27a-5p, regulated by TGF-β1 signaling in neuroblastoma models (44). In ovarian cancer, the cytotoxicity of NK cells is impaired by miR-20a, which is known for targeting MICA/B. MICA/B are ligands that bind to NKG2D receptors on NK cells for the recognition and elimination of cancer cells (45). Furthermore, NK cells can secrete miRNAs to modulate the TME and assert their effector functions. Interestingly, NK cell-derived exosomes containing miR-186 was shown to prevent TGF-β1-dependent inhibition of NK cells, thus reducing neuroblastoma tumors (46).

These immune cells and the miRNAs that regulate them highlight their critical contributions towards the modulation of the immune TME by miRNAs (**Table 1**). The consequences of miRNA mediated immune modulation towards tumor destruction or escape reveals their potential as therapeutic targets in immunotherapeutic strategies.

**Table 1:** Summary of miRNA functions in the TIME.

Immune TME cell	MiRNAs	Target genes	Functions	Cancer	Ref.
CD8+ CTLs	miR-23a-3p miR-19a-3p miR-183/96/182 (m96cl) miR-200c	Pten Slc6a8 Foxf2, Zeb1 Zeb1	T cell exhaustion and apoptosis Immune evasion Inhibition of tumor growth and metastasis Stimulation of CD8+ CTLs	HCC AML NSCLC Melanoma	(14) (15) (16) (17)
Tregs, MDSCs	miR-208b, miR-21 miR-26b-5p miR-1298-5p miR-200c	Pdcd4 Pten Msh2 Cd274	Resistance to oxaliplatin, proliferation Expansion and activation of MDSCs Proliferation Reduced immunosuppresion	CRC, Glioma ESCC Glioma CRC	(22–24) (26) (27) (47)
DCs	miR-21, miR-29 miR-203	Tlr7/8 Tlr4	Protumoral inflammation, tumor growth and metastasis Impaired antigen presentation, tumor growth	LLC PDAC	(8) (30)
TAMs	miR-183-5p, miR-222-3p, miR-142-5p miR-29a-3p, miR-21-5p miR-182	Pten Stat3 Tlr4	M2 polarization, tumor growth Immunosuppression M2 polarization	Breast Ovarian Breast	(32) (34) (48)
B cells	miR-330-3p	Jam2	Migration and invasion	Ovarian	(36)
NK cells	miR-27a-5p miR-186	Cx3cr1 Tgfbr1/2	Reduced NK infiltration Increased NK cell cytotoxicity	Neuroblastoma	(44) (46)

## Targeting the immune TME with miRNA therapeutics

Several miRNA-based therapies have entered clinical stage testing. TTX-MC138, a proprietary compound from *Transcode Therapeutics*, is an inhibitor of miR-10b and has shown considerable therapeutic efficiency in pre-clinical models. MiR-10b is a critical mediator of cell migration, invasion, and metastasis initiation (49, 50). It also targets MICB to escape the rejection of malignant cells by NK cells (51). Thus, inhibiting miR-10b can modulate the antitumor immune response. The peptide-oligonucleotide conjugate Pep-21, consisting of an anti-miR-21 and a PDL1-binding peptide was recently shown to target both macrophages displaying PD-L1 and tumor cells. Pep-21 efficiently neutralized miR-21 expression, in both cellular components, which led not only to the suppression of tumor progression and invasion, but also to the reprogramming of M2-like macrophages towards an M1-like phenotype. The modulation of macrophages by Pep-21 resulted in the inhibition of angiogenesis and the promotion of an antitumor response in the TME (52). Sahraei et al. (2019) demonstrated that miR-21-deficient TAMs present an inflammatory gene signature. On the other hand, antagonizing miR-21 was found to increase levels of granzyme B and degranulation of CD8+ T cells. Consequently, miR-21-deficient TAMs enhanced the cytotoxicity of CD8+ T cells in the immune TME. Notably, this study also highlighted the capacity of the anti-miR-21 regimen to efficiently reduce the growth of tumors harbouring miR-21-deficient cancer cells (53). This observation suggests that the antitumor response relies on the inhibition of miR-21 not only in cancer cells, but also in other components of the immune TME, such as macrophages. Together, these studies strongly support the development of miR-21 inhibitors as novel anti-cancer treatments and as immunomodulators.

TAMs are widely present in many tumors. The conditional knockout of miR-182 in macrophages highlighted the ability of a miRNA to interfere with the pro-tumoral polarization of M2 TAMs, in the immune TME. TAM-specific or systemic depletion of miR-182 resulted in reduced breast cancer growth and was associated with the decreased expression of M2-like markers. Treatment of murine breast tumors with the antagomir-182 proved effective at reprogramming TAMs and inhibiting tumor growth (48). Furthermore, it was found that the administration of modified tumor-derived extracellular vesicles loaded with miR-142 and let-7i increases mature DCs, Th1 and cytotoxic T cells in a murine model of triple-negative breast cancer. This was also associated with an augmentation of IFN-γ production and release of granzyme B (54). In a study investigating the mechanism underlying the increased radiosensitivity of HPV-positive head and neck squamous cell carcinomas (HNSCC) compared to HPV-negative HNSCCs, the upregulation of miR-9 was observed and raised interest for its potential application as a therapeutic target for the treatment of HPV+ HNSCC. Experiments using miR-9 mimics have demonstrated that the induction of M1-like macrophage polarization was responsible for the heightened radiosensitivity (55).

Tumor infiltrating lymphocytes (TILs) represent important components of the immune TME. In an orthotopic murine neuroblastoma model, the administration of miR-186 enhances the cytotoxic potential of NK cells by suppressing the TGF-β1 signaling pathway through TGF-βR1/2, which led to the restoration of NKG2D and DNAM-1 expression on NK cells (46). MiR-155 is another promising therapeutic target. Recently, the ability of miR-155 to enhance CD8+ T cell antitumoral function has been described. Through the silencing of the Akt inhibitor Ship1, miR-155 was found to indirectly stimulate the activity of the Polycomb repressor complex (PRC2), which has been reported to be critical in preventing T cells exhaustion and senescence (56).

Various reports have shown that miRNAs can also be used in combination with other therapeutic modalities to improve their antitumoral effects and reduce resistance to therapy. A good example is, a miRNA targeting PD-L1. In a recent preclinical colorectal cancer model, miR-200c was used in combination with α-PD-L1 checkpoint inhibitor and the BRAF inhibitor, Dabrafenib, to further enhance the antitumor immune response. This triad was shown to act synergistically and enhance the immunogenicity of cell death, which resulted in the reduction of Tregs and PD-L1 expression on cancer cells. This combination potentiated tumor eradication, while using a much lower dose of each therapeutic agent individually (47).

Altogether, these preclinical and clinical miRNA candidates highlight their role as immunomodulators of the TME and their therapeutic promise. Interestingly, macrophages appear to be important targets for miRNA regulation. The inhibitor of miR-182 was delivered precisely to macrophages by being packaged into modified vesicles that trigger phagocytosis. It is therefore possible to direct miRNA therapeutics towards phagocytic cells and, therefore, possibly towards other immune cell subsets.

## Current delivery methods of miRNA and their limitations

The most significant hurdle to the clinical development of miRNA therapies is their delivery to the tumor bed to exert maximal *in situ* effect. There are many available technologies enabling such a delivery, but their efficacy is limited. Moreover, the limited capacity to chemically improve the stability of the RNA interfering molecules without hindering their regulatory function has led to the development of multiple encapsulation strategies. These include liposomes and polymer-based nanoparticles, inorganic vectors made of calcium phosphate, porous silica, gold, carbon, or carbonate apatite, nanotechnologies such as bacterial minicells and extracellular vesicle-based vectors (2, 57). These have been reviewed extensively elsewhere (58).

Despite these innovations, the efficiency of miRNA delivery systems remains suboptimal. The lack of blood perfusion in tumors, the phagocytic action of immune cells, endosomal entrapment and TME hostility, which limits the uptake of miRNAs by cancer cells, explains miRNA delivery limitations. Moreover, the chemical modification of miRNAs and the usage of exogenous vehicles have been shown to generate immune toxicities. Ultimately, the lack of tumor specificity, essential to maintaining a low toxicity profile and reduce off-target effects, remains an impediment to the safety and efficacy profile of potential miRNA therapeutics (3). For example, the construct of TargomiR, a candidate for refractory malignant pleural mesothelioma, consists of minicells loaded with miR-16 mimics and coated with anti-EGFR antibodies meant to enhance tumor specificity. In a phase I clinical trial designed to evaluate its safety profile, significant adverse reactions were reported. Although the toxicity profile was subsequently judged to be acceptable, propelling TargomiR into phase II clinical trials, these safety issues require close monitoring and follow up (59). Other miRNA therapies have been rejected. The phase I clinical trial for MRX34, a miR-34a mimic delivered in liposomal nanoparticles has been withdrawn due to a severe immune toxicity in five patients afflicted with cancers of different origins, which ended in mortality for four of them. Since the liposomal vector has been successfully administered in other clinical trials, it is though that the miR-34a mimic was responsible for these severe adverse events (60). Such drawbacks could potentially be alleviated using viral delivery vectors. Therefore, we suggest the use tumor-targeted OVs to deliver miRNA with minimal harm towards healthy cells (61).

## Delivering therapeutic miRNAs with OVs

OVs are immunotherapeutic agents capable of selectively infecting and lysing cancer cells, while eliciting antitumor immune responses. They can target cancer cells and the tumor stroma, through the exploitation of aberrant and oncogenic pathways. Their mechanisms of action, including oncolysis, antitumor immunity, vascular disruption, and transgene expression have been extensively reviewed elsewhere (5, 62–64). Considering the potential impact of these mechanisms on therapeutic outcome, optimizing these attributes is the subject of extensive research. In addition to their capacity to remodel the immune TME, OVs have been used to deliver genetically encoded transgenes directly into the TME. The untapped potential of miRNA therapy exemplified through the lack of efficient carrier systems coupled with the possibility of engineering OVs capable of delivering miRNAs directly in the TME encouraged researchers to develop OVs expressing such RNA interfering molecules. The stability provided by OVs harbouring miRNAs is inferior to those provided by encapsulation strategies. However, the insertion of multiple copies of the desired miRNA, into the viral genome, could help to overcome this limitation (65).

When designing OVs to express functional miRNAs, it is crucial to carefully select the exact sequence to be inserted into the viral genome, as it will affect the production of mature miRNA molecules. Mature miRNAs are generated by precursor and primary intermediates that are processed by the cellular machinery to express functional interfering RNA molecules. Therefore, both pre-miRNA and pri-miRNA sequences can be used to express the desired miRNA molecule, but with different efficiency. In 2020, Brachtlova et al. engineered oncolytic adenoviruses to express miR-1 and miR-26b. The former was chosen because of its low expression in many cancer cells and tumor-suppressor functions, while the latter improved adenoviral propagation in prostate cancer cells. They compared the expression of mature miRNAs from adenoviruses encoding pre-miRNA and pri-miRNA constructs. The endogenous pre-miRNA sequence was flanked by 120 nucleotides to produce the pri-miRNA. Their results show that pri-miRNA-expressing adenoviruses produced far greater quantities of mature miRNAs than those expressing pre-miRNAs. The adenoviral miR-1 and miR-26b were then able to silence validated target genes. Altogether, they showed that pri-miRNA transgene could be more efficiently expressed by a double-stranded DNA virus and regulate gene expression, providing a template for future similar endeavours (66).

## Improving viral oncolysis with miRNAs

Above all strategies used by OVs, viral oncolysis remains its top mechanism to destroying cancers. A study by Rovira-Rigau et al. revealed that miR-99b and miR-485 could enhance adenoviral oncolysis by facilitating virion production. The oncolytic adenovirus ICOVIR15 was armed with either miR-99b or miR-485 and the resulting virus proved fitter and more antitumorigenic in PDAC cell lines. This was attributed to the targeting of ELF4, MDM2, and KLF8 genes that are involved in the regulation of E1A and late viral protein expression. Therefore, this enhanced viral oncolysis. In a subcutaneous model of human PDAC cell line implanted in nude mice, ICOVIR15 expressing miR-99b or miR-485 significantly reduced tumor growth, when compared to parental ICOVIR15 (67). Furthermore, an oncolytic adenovirus was engineered to express a miR-222 sponge (AdNuPAR-E-miR222-S) and was able to reduce tumor growth in an *in vivo* model of PDAC. AdNuPAR-E-miR222-S effectively inhibited miR-222 expression and upregulated downstream target genes. This enhanced both adenoviral replication and cytotoxicity in preclinical models of PDAC (68). Moreover, the upregulation of miR-222 in PDAC and its ability to limit adenoviral propagation highlighted the miR-222 sponge as a promising therapeutic candidate.

Recently, the oncolytic vesicular stomatitis virus dM51 (VSVd51) was also modified to express a carefully selected artificial miRNA and enhance its antitumoral properties (6). Using a high throughput method, the authors were able to select miRNAs capable of significantly improving viral oncolysis, ultimately pursuing the most interesting candidate. Such a strategy inherently optimizes the efficiency of the transgenic modification but could perhaps restrain its therapeutical range. However, the modified OV was tested successfully in various tumor models of different tissue origins. Tailoring the enhancement of OV by miRNA to the molecular landscape of targeted tumors could pave the way for the personalization of OV therapy. In this case, the artificial miR-4 was shown to target pathways involved in epigenetic regulation, cytoskeletal dynamics, and resistance to OVs. This translated into improved tumor control and survival of mice treated with VSVd51 expressing amiR-4. Interestingly, the authors also showed that the artificial miRNA could be transmitted by infected cells to neighbouring uninfected cells *via* SEVs. These results suggest that miRNAs expressed by OVs can remodel the molecular landscape of the TME through their propagation to uninfected cells, stromal cells, and other cancer-associated components. By disseminating in the TME, the miRNA can reach areas or cell types resistant to OVs to supplement the impact of viral oncolysis on the tumor. It may also sensitize a wider range of malignant and malignant-associated cells to its viral carrier. The selective delivery of miRNA therapeutics to the tumor site remains a significant challenge. OVs enable such a precise delivery, which reduces the potential for off-target effects. The demonstration that OV-expressed miRNAs can travel throughout the TME in SEVs reinforces the concept that these miRNAs can have a strong impact on gene expression and OV therapy. This study emphasizes the importance of selecting a miRNA that is compatible with the viral platform to optimize the synergies of the combination therapy.

As discussed above, the selection process is of crucial importance. It would be prudent to consider the effect of OV therapy on the expression of endogenous miRNA and on overall gene expression. The miRNA supplementation by miRNA-expressing OVs does not occur in untreated tumors, but rather in actively infected tumors. This can significantly modify the TME and change its susceptibility to various miRNAs. Vazifehmand et al. recently demonstrated that HSV-G47d upregulated the expression of several miRNAs involved in the pathogenesis of glioblastoma, therefore revealing previously hidden therapeutic targets (69). In another study, Chen et al. analyzed the differential expression of miRNAs following Newcastle disease virus (NDV) infection of HeLa cells (70). They identified several miRNAs differentially expressed and involved in tumor progression as well as tumor suppression. Many genes regulated by these miRNAs are associated with immune functions suggesting that NDV can modulate the antiviral response by engaging miRNA pathways. The authors also observed that miR-4521, one of the differentially expressed miRNA, negatively affected the replication of NDV. Comparing these two studies, while Wedge et al. utilized miRNAs that favoured the replication of OVs, Chen et al. identified miR-4521 as an impediment to OV replication and therefore, as a potential therapeutic target (6, 70). The inflammatory response induces a miRNA response that could also be targeted therapeutically to enhance OV therapy. The miRNAome is significantly altered by OV infection and the immune response that is induced translates into differentially expressed miRNAs acting as immune modulators. Therefore, these studies support the optimal design of OVs expressing miRNAs to maximize their efficacy. As OVs appear to activate pro- and/or anti-inflammatory miRNA programs, OVs can be rationally designed to further modulate the immune TME by expressing miRNAs.

In another study, the oncolytic herpes simplex virus (HSV) remodeled the immune TME by diminishing the presence of anti-inflammatory TAMs and increasing the percentage of TILs including Th1, cytotoxic CD8+ T and NK cells (71). Macrophages represented the dominant immune cell population, as they accounted for more than half of the immune infiltrate. Furthermore, single-cell RNA-seq (scRNA-seq) analyses revealed seven distinct macrophages clusters. Following HSV treatment, immunosuppressive macrophage clusters characterized by elevated expression of Mrc1 and Il-10 were diminished and clusters grouping pro-inflammatory macrophages expressing high levels of MHC class II transcripts were increased. Therefore, this demonstrated a phenotypical switch of the heterogenous TAM population. TILs, including CD8+ T and NK cells also exhibited a pro-inflammatory phenotypical switch characterized by increased expression of Gzmb and Prf1.

Altogether, miRNAs can be used to effectively improve viral oncolysis (**Table 2**). As we have discussed here, a lot can be done to optimize the selection of miRNAs to be inserted into the genome of OVs, from the design of high-throughput experiments aimed at identifying relevant miRNAs or characterizing the effects of OVs in the targeted tumors, to carefully engineering the miRNA transgene. Considering the tremendous impact of immunotherapies on cancer treatment, improving the ability of OVs to remodel the immune TME can ultimately translate into better clinical outcomes. MiRNAs have the potential to enable such an improvement. MiRNAs can be added to OVs to successfully improve viral oncolysis and provide direct tumor suppressive and immune TME remodeling functionalities.

**Table 2:** Summary of OVs expressing miRNAs and their mechanisms of action.

OV	MiRNAs	Target genes	Effects	Cancer model	Ref.
Adenovirus	miR-26b	Ptgs2	Improved viral propagation	Prostate	(66)
Adenovirus (ICOVIR15)	miR-99b miR-485	Elf4 Mdm2	Increased viral replication Reduced tumor growth	PDAC	(67)
Adenovirus (AdNuPAR-E-miR222-S)	miR-222 inhibitor	miR-222	Increased viral replication and cytotoxicity	PDAC	(68)
VSVd51-amiR-4	amiR-4	Arid1a	Improved viral oncolysis	PDAC Melanoma	(6)
Adenovirus (AdCN205)	miR-34a	Bcl2 Sirt1	Inhibited proliferation Repressed metastasis and angiogenesis Sensitized cancer cells to IL-24 Improved viral cytotoxicity	HCC	(72)

These studies demonstrate the efficacy of OVs as transporter of miRNA cargo and highlight their capacity to regulate gene expression *in situ*, while limiting off-target effects. Although modestly documented, another equally promising avenue is the potential synergistic combination of OVs and miRNA therapy to target the immune TME and enhance the immunomodulatory properties of OVs.

## Remodeling the immune TME with OVs expressing miRNAs

Lou et al. published a study discussing the therapeutic efficacy of AdCN205, an engineered oncolytic adenovirus expressing both miR-34a and IL-24. By targeting, Bcl2, an antiapoptotic gene, and Sirt1, the ectopic expression of miR-34a inhibited the proliferation of tumor cells and induced apoptosis. It also simultaneously repressed metastasis and angiogenesis. The inhibition of Bcl2 sensitized tumor cells to IL-24, a cytokine known to exert an antitumor effect. Most importantly, the tumor selectivity of AdCN205 led to increase *in vivo* cytotoxicity of cancer cells, while preserving healthy counterparts (73). MiR-34a also regulates the number of PD-LI-expressing TAMs and is associated with a higher frequency of Th1 and CD8+ naïve T cells (72). Thus, antitumor immunity could further explain the observed tumor regression and provide evidence to support the development of OVs expressing immunomodulatory miRNAs.

Additionally, miR-99b was used to improve the tumoral fitness of the oncolytic adenovirus ICOVIR15, along with miR-485. This was described as underlying mechanism of tumor reduction. However, in a study by Wang et al., miR-99b was shown to reprogram TAMs towards an antitumor phenotype (74). Therefore, considering the important role of TAMs in the immune TME, this could also explain part of the efficacy of ICOVIR15. Another study by Brachtlova et al., armed a similar virus with miR-26b (66). Its depletion is reportedly associated with poor survival in HCC. MiR-26b also upregulates the secretion of TNF-α, IFN-γ, IL-6, and IL-2 in CD4+ and CD8+ T cells, to enhance T cell responses and inhibit tumorigenesis (75). An OV expressing miR-26b could therefore activate a T cell response more conducive to tumor eradication compared to its non-miR expressing viral controls.

**Figure 1:**
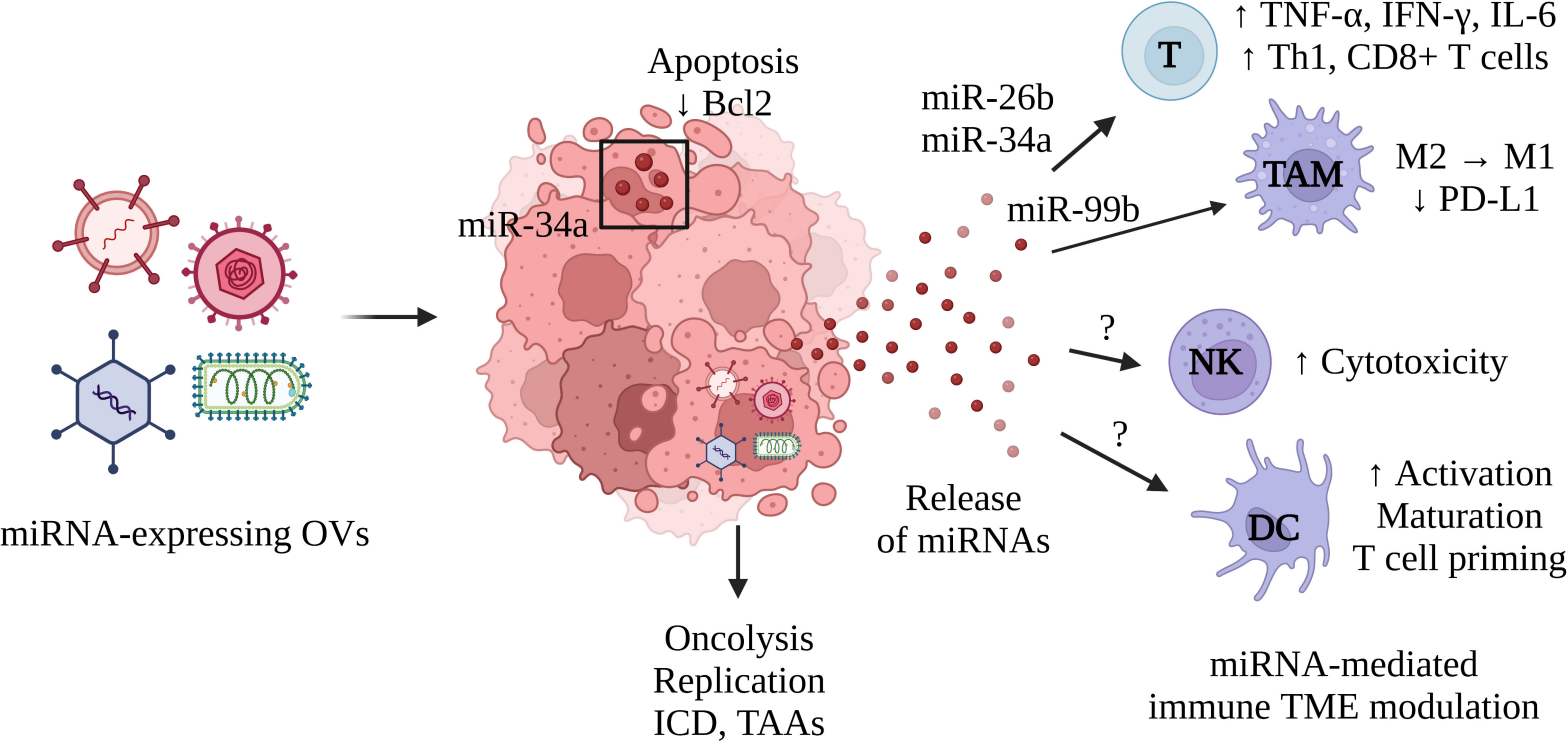
Schematic representation of the immune TME remodeling induced by miRNA expressed from OVs. OVs expressing miRNAs infect cancer cells and produce miRNAs capable of spreading across the TME and modulating immune function. This process can be used to improve the function of effector T cells, stimulate a Th1 response, activate CD8+ T cells, or to favor an M1-like phenotype in TAMs. This mechanism could be leveraged to stimulate the effector functions of NK cells or to recruit and activate DCs. MiRNAs can also regulate gene expression in infected cancer cells and increase their immunogenicity, by inducing cell death or altering their immune profile. Oncolysis enables viral replication as well as the release of tumor-associated antigens (TAAs). This process is characterized by an immunogenic form of cell death termed immunogenic cell death (ICD) and the immunomodulatory molecules it produces (ecto-calreticulin, adenosine triphosphate, high mobility group box 1, etc.). T, T cells; TAM, tumor-associated macrophages; NK, Natural killer cells, DC, Dendritic cells. Created with BioRender.com.

We have already discussed the regulation of TAMs, Th1, and CD8+ T cells by AdCN205-expressed miR-34a (73). In T cells, miR-34a targets multiple genes involved in processes such as T cell migration and TCR signaling, according to a study published by Hart et al. (76). This article highlighted the immunomodulatory function of miR-34a but provided contradicting evidence supporting a pro-tumoral effect in T cells. The overexpression of miR-34a led to decreased secretion of perforin and expression of CD11A, involved in T cell migration, adhesion, and co-stimulation. Nonetheless, the available data showing AdCH205-miR-34a effectively decreased tumor growth, suggests that miR-34a can remodel the immune TME. A miR-222 sponge was also integrated into the genome of an oncolytic adenovirus and resulted in increased replication and cytotoxicity leading to the inhibition of tumor growth in a PDAC model (68). MiR-222 is, however, also involved in immune evasion (77). Therefore, interfering with such a process could have revealed the tumor to the immune system, restoring its immunogenicity, and thus promoting an antitumor immune response. Altogether, these studies demonstrate the potential for miRNAs expressed by OVs to modulate immune responses and remodel the immune TME (**Figure 1**).

## Perspectives

As our understanding of the molecular landscape of tumors and the remodeling of the TME after OV treatment grows through high-throughput analyses, engineering OVs with optimally selected miRNAs to maximize the efficacy of the therapy becomes more accessible. In a clinical setting, tumor biopsies or resections could be analyzed to reveal tumor susceptibilities in the form of dysregulated miRNAs, genes, or pathways to be exploited and OVs expressing the selected miRNAs could be quickly produced. This would require a deep understanding of the relationship between OVs, miRNAs, and the molecular phenotype of tumors. To our knowledge, there is a lack of molecular profiling studies on tumors treated with OVs expressing miRNAs. This would provide a thorough description of the impact of miRNAs on OV therapy. More work is also required to better understand the life cycle of the OV-produced miRNA and its mechanism of action. Throughout this review, we presented studies describing various OVs expressing only one miRNA. Future research will certainly work towards developing OVs capable of expressing multiple miRNAs cooperatively. OV-miR therapy can also be combined with other therapeutic modalities. Indeed, OVs are often combined with radiotherapy, immune checkpoint inhibitors (ICIs), CAR T cells, or small molecule modulators (64, 78–80). MiRNAs could therefore be selected to work synergistically with existing and emerging precision therapies.

## Conclusion

The ability of miRNAs to regulate the immune TME has been well described, yet miRNA-based therapies face several challenges for clinical translation, including the requirement for an adequate *in vivo* delivery system. OVs can be engineered to express rationally selected miRNAs capable of improving viral replication and oncolysis and remodel the immune TME into which they are specifically delivered. The miRNAs can effectively regulate the expression of target genes, remodel the immune TME, and travel across the TME through SEVs. Combining miRNA therapeutics and OVs represent an emerging synergistic platform to overcome pre-existing miRNA limitations and enhance immunomodulation of the TME. This has the real potential to impact cancer patient prognosis.

## Author contributions

GSC, DP, HG, LD, AAA and LHT conceived, wrote and revised the manuscript. LHT supervised the manuscript. All authors contributed to the article and approved the submitted version.
